# Multicenter derivation and validation of an early warning score for acute respiratory failure or death in the hospital

**DOI:** 10.1186/s13054-018-2194-7

**Published:** 2018-10-30

**Authors:** Mikhail A Dziadzko, Paul J Novotny, Jeff Sloan, Ognjen Gajic, Vitaly Herasevich, Parsa Mirhaji, Yiyuan Wu, Michelle Ng Gong

**Affiliations:** 1 Department of Anesthesiology and Perioperative Medicine, Mayo Clinic, Rochester, MN USA; 2 Department of Anesthesiology, HCL CHU Croix-Rousse, Lyon, France; 3 Department of Health Sciences Research, Mayo Clinic, Rochester, MN USA; 4 Department of Pulmonary and Critical Care Medicine, Mayo Clinic, Rochester, MN USA; 5 Department of Systems & Computational Biology, Montefiore Health System, Albert Einstein College of Medicine, Bronx, NY USA; 6 Division of Critical Care Medicine, Department of Medicine, Montefiore Health System, Albert Einstein College of Medicine, Main Floor, Gold Zone, 111 East 210th Street, Bronx, NY 10467 USA

**Keywords:** Acute respiratory failure, Prediction, Electronic health records, Early warning scores, Random forest

## Abstract

**Background:**

Acute respiratory failure occurs frequently in hospitalized patients and often starts before ICU admission. A risk stratification tool to predict mortality and risk for mechanical ventilation (MV) may allow for earlier evaluation and intervention. We developed and validated an automated electronic health record (EHR)-based model—Accurate Prediction of Prolonged Ventilation (APPROVE)—to identify patients at risk of death or respiratory failure requiring >= 48 h of MV.

**Methods:**

This was an observational study of adults admitted to four hospitals in 2013 or a fifth hospital in 2017. Clinical data were extracted from the EHRs. The 2013 patients were randomly split 50:50 into a derivation/validation cohort. The qualifying event was death or intubation leading to MV >= 48 h. Random forest method was used in model derivation. APPROVE was calculated retrospectively whenever data were available in 2013, and prospectively every 4 h after hospital admission in 2017. The Modified Early Warning Score (MEWS) and National Early Warning Score (NEWS) were calculated at the same times as APPROVE. Clinicians were not alerted except for APPROVE in 2017cohort.

**Results:**

There were 68,775 admissions in 2013 and 2258 in 2017. APPROVE had an area under the receiver operator curve of 0.87 (95% CI 0.85–0.88) in 2013 and 0.90 (95% CI 0.84–0.95) in 2017, which is significantly better than the MEWS and NEWS in 2013 but similar to the MEWS and NEWS in 2017. At a threshold of > 0.25, APPROVE had similar sensitivity and positive predictive value (PPV) (sensitivity 63% and PPV 21% in 2013 vs 64% and 16%, respectively, in 2017). Compared to APPROVE in 2013, at a threshold to achieve comparable PPV (19% at MEWS > 4 and 22% at NEWS > 6), the MEWS and NEWS had lower sensitivity (16% for MEWS and NEWS). Similarly in 2017, at a comparable sensitivity threshold (64% for APPROVE > 0.25 and 67% for MEWS and NEWS > 4), more patients who triggered an alert developed the event with APPROVE (PPV 16%) while achieving a lower false positive rate (FPR 5%) compared to the MEWS (PPV 7%, FPR 14%) and NEWS (PPV 4%, FPR 25%).

**Conclusions:**

An automated EHR model to identify patients at high risk of MV or death was validated retrospectively and prospectively, and was determined to be feasible for real-time risk identification.

**Trial registration:**

ClinicalTrials.gov, NCT02488174. Registered on 18 March 2015.

**Electronic supplementary material:**

The online version of this article (10.1186/s13054-018-2194-7) contains supplementary material, which is available to authorized users.

## Background

At 784 cases per 100,000 hospitalizations, acute respiratory failure (ARF) is one of the most common acute organ failures in the hospital [1] and is associated with a 6-month mortality of 30%, increased hospital readmission, and functional impairment among survivors [2–4]. Clinical deterioration in ARF can be seen 8–48 h prior to critical care intervention [5]. Yet delayed recognition of clinical deterioration is common, and is associated with worse outcomes [6]. Failure to recognize developing respiratory failure is the most common reason for delayed Rapid Response Team (RRT) activation which has been associated with increased in-hospital mortality [7, 8].

Most early warning systems (EWS), like the Modified Early Warning Score (MEWS) or the National Early Warning Score (NEWS), predict ICU admission, cardiac arrest, or death, as an indication of clinical deterioration [9, 10]. Cardiac arrest and death are infrequent and represent the late, end result of organ dysfunction. ICU admission depends as much on hospital-level factors as patient condition. However, respiratory failure requiring mechanical ventilation (MV) is a widely accepted criterion for ICU admission and is consistently associated with increased mortality and morbidity [11]. A multicenter clinical model to predict for acute respiratory failure, in addition to death, may be able to identify acutely ill patients in a variety of hospitals earlier in the course of their critical illness, when prompt interventions may be better able to affect the likelihood or duration of MV, ICU admission, morbidity, death, or unwanted life-sustaining therapies.

The goal of this study is to develop and validate a multicenter, EHR-based risk stratification tool to identify patients at high risk of death or acute respiratory failure requiring MV that is prolonged for 48 h or beyond.

## Methods

### Study design and setting

The APPROVE (Accurate Prediction of Prolonged Ventilation) score was derived and validated retrospectively on patients from four hospitals in a 2013 cohort and then externally validated prospectively in a different hospital in 2017. As a comparison, the MEWS and NEWS were also calculated in the validation cohorts in 2013 and 2017 at the same time points when APPROVE was calculated. All study hospitals have a comprehensive EHR system with a data warehouse.

The study protocol was reviewed and approved by each hospital’s Institutional Review Boards. Informed consent was waived.

### Study population

The 2013 cohort consisted of adult (> age 18 years) admissions in 2013 to four hospitals within two tertiary academic centers: Montefiore Moses division and Jack D. Weiler Hospital in the Montefiore Health System (Bronx, NY, USA); and St Mary’s and Rochester Methodist at the Mayo Clinic (Rochester, MN, USA). Each admission was randomly assigned 50:50 to either the derivation cohort for model development or the internal validation cohort for model verification. The 2017 external validation cohort consisted of all adult admissions to Wakefield Hospital in the Montefiore Health System from January 17 to March 31, 2017. In both cohorts, patients who were mechanically ventilated prior to admission were excluded and multiple hospitalizations per patient were included.

### Data collection

Only variables that are routinely collected for clinical care were included as candidate variables for the derivation of APPROVE. This included baseline demographics, vital signs, laboratory data, and hospital interventions/assessments (see Additional file 1: Table S1). A site-specific data dictionary was created for each hospital in order to unify the collection of data. Each data element in the dictionary was mapped to an existing field at each hospital EHR, and site-specific abstraction and validation procedures were developed. Two different EHR systems were used in the four hospitals in the 2013 cohort and a different third EHR vendor was used in the prospective 2017 cohort.

### Outcomes

A composite qualifying event was defined as death in the hospital or intubation and subsequent MV for 48 h or longer. In the EHR of the study hospitals, all patients on MV can be identified but the reason for intubation cannot. Most intubations were short term and reflected elective intubation for surgery, procedures, or milder forms of respiratory failure with lower mortality and morbidity. For those reasons, we did not consider mechanical ventilation < 48 h without death to be a qualifying event. Time of event was defined as the time of death or initiation of MV that lasted at least 48 h, whichever occurred first.

### Model development

At multiple, randomly selected time points over the hospital stay of each patient in the 2013 derivation cohort, the closest EHR data collected prior to that time point were used in the derivation model. A patient was considered to have a qualifying event if the event occurred within the next 48 h of the selected time point. The qualifying event was a binary variable. However, in the model we used it as a continuous dependent variable so that the predicted values would be a continuous likelihood estimation score from 0 to 1. The random forest (RF) was implemented using the “randomForestSRC” R-package (http://cran.r-project.org/web/packages/randomForestSRC/). Random forest models are machine learning algorithms. They fit multiple classification and regression trees (CART) on random subsets of the patients and random subsets of the variables. CART models start by including the one variable that is the best predictor of the outcome, and then sequentially add additional variables one at a time until no other variables improve the prediction. These multiple models are combined using maximum likelihood methods to create the final estimates. Missing data were imputed using a random forest algorithm to match patients having known values that are most similar to other patients with the missing values at the same time point. This method is pattern based, as opposed to more commonly used arbitrary imputation (e.g., fixed, average, best, or worst value imputation).

Variable importance, minimal tree depth of each variable, error rates (misclassification), and predicted values were obtained, and a maximum likelihood estimator (MLE) was calculated and used as the APPROVE score. Variable importance is a measure of how changes in a variable affect the model prediction. The minimum depth is the earliest the variable is included in any of these multiple CART trees. Higher values of the APPROVE score are associated with greater likelihood of an event. The area under the receiver operator curve (AUROC) was used to measure the discrimination of the model; performance was evaluated by calculating sensitivity, specificity, and positive and negative predicted values (PPV and NPV) (see Additional file 1: Table S2).

### Model validation

The model performance was first evaluated in the retrospective, internal validation 2013 cohort. To mimic how APPROVE may be used prospectively in clinical practice, the APPROVE score was calculated every time there was a data point in the 2013 validation cohort. Each patient was examined to determine the first time a score exceeds a certain cutoff, if ever, and then evaluated for an event at any subsequent time. To determine the performance of APPROVE, three cutoff values (0.15, 0.20, 0.25) were selected based on the derivation cohort (see Additional file 1: Table S2). As a comparison, at each time point that APPROVE was calculated, the MEWS and NEWS were also calculated using the same data.

APPROVE was also validated prospectively at the external 2017 hospital. Real-time calculation of APPROVE whenever new data were available was not possible because of the technical and computational demand of real-time data extraction and triggering of score calculation. Instead, APPROVE was calculated every 4 h (00:00, 04:00, 08:00, 12:00, 16:00, 20:00) for all adult patients after admission to the hospital using the last available data. In all calculations of APPROVE in 2013 and 2017, missing data were replaced by the imputed values determined from the 2013 derivation cohort. For calculating the NEWS and MEWS, last available values were pulled forward. If values were missing, they were assumed normal.

As APPROVE was not calculated until after hospital admission, patients who presented with or developed the primary event of interest (respiratory failure requiring MV or death) in the emergency department (ED) prior to hospital admission, and calculation of any APPROVE score, were excluded from the analysis. Clinicians could not see scores for their patients. However, clinicians were alerted if their patient on the hospital wards had a score > 0.25.

Calculation of scores and analyses were performed using R version 3.1.1 (www.R-project.org), SAS version 9.4, and JMP 11.1.1 (SAS Institute Inc., Cary, NC, USA).

## Results

Clinical data were abstracted from 68,775 admissions in 2013 for the retrospective derivation and internal validation cohort. In the 2017 cohort, clinical data were abstracted in the same manner from 2258 adult admissions to the Wakefield Hospital after excluding 33 patients because they presented with or had the event in the ED prior to the calculation of any APPROVE score. The hospital mortality for patients on mechanical ventilatio >= 48 h was 33% in both 2013 and 2017 compared to mortality rates of 1.4% in 2013 and < 1% in 2017 for patients with MV < 48 h.

### Study population characteristics

The baseline demographics, vital signs, and laboratory findings of the 2013 and 2017 cohorts are presented in Table 1. The event rate was higher in 2013 at 3% compared to 2017 at 1.6%, likely because events that developed before any calculation of APPROVE which was scheduled every 4 h were excluded from the 2017 cohort (total event rate of 3.0% in the 2017 hospital, same as in 2013, if those patients were included). There were fewer missing data in the prospective cohort but otherwise the values were similar.

**Table 1:** Characteristics and variables used to calculate APPROVE score in each cohort

	Retrospective 2013 cohort				Prospective 2017 cohort	
Variable	Fitting cohort (*N* = 34,387)	Validation cohort (*N* = 34,388)	Total (*N* = 68,775)	Number (%) missing	Validation cohort (*N* = 2258)	Number (%) missing
Qualifying event, yes	958 (3%)	1072 (3%)	2030 (3%)	0 (0%)	35 (1.5%)	0 (0%)
Demographics						
Age	57.8 ± 19.5	58.0 ± 19.5	57.9 ± 19.5	0 (0%)	55.9 ± 20.4	0 (0%)
Body mass index	29.6 ± 9.0	29.6 ± 8.9	29.6 ± 9.0	12,595 (18%)	31.3 ± 9.2	146 (6.4%)
Height (cm)	165.8 ± 12.8	165.6 ± 13.0	165.7 ± 12.9	11,900 (17%)	163.4 ± 10.4	136 (6.0%)
Sex, female	20,452 (59%)	20,595 (60%)	41,047 (60%)	0 (0%)	1658 (67%)	0 (0%)
Weight (kg)	81.6 ± 26.9	81.4 ± 26.9	81.5 ± 26.9	7907 (11%)	83.4 ± 27.7	99 (4.4%)
Medication use (yes)^*^						
Dobutamine	24 (0%)	22 (0%)	46 (0%)	0 (0%)	0	0 (0%)
Dopamine	20 (0%)	20 (0%)	40 (0%)	0 (0%)	0	0 (0%)
Epinephrine	6 (0%)	3 (0%)	9 (0%)	0 (0%)	0	0 (0%)
Norepinephrine	165 (0%)	137 (0%)	302 (0%)	0 (0%)	4	0 (0%)
Vasopressin	17 (0%)	13 (0%)	30 (0%)	0 (0%)	2	0 (0%)
Oxygen requirement, FiO_2_	0.5 ± 0.2	0.5 ± 0.2	0.5 ± 0.2	0 (0%)	0.5 ± 0.3	0 (0%)
RASS	−0.8 ± 1.5	−0.8 ± 1.5	−0.8 ± 1.5	59,105 (86%)	−0.89 (1.76)	2245 (99.3%)
Oxygen devices				0 (0%)		0 (0%)
Room air (or no device)	30,677 (89%)	30,557 (89%)	61,234 (89%)		1684 (68%)	
Flow/hood/tent/isolette/mask	76 (0%)	81 (0%)	157 (0%)		8 (0%)	
Low-flow nasal cannula	3409 (10%)	3535 (10%)	6944 (10%)		650 (26%)	
High-flow nasal cannula	7 (0%)	6 (0%)	13 (0%)		17 (1%)	
Nonrebreather mask	66 (0%)	63 (0%)	129 (0%)		31 (1%)	
CPAP, AVAPS, or BIPAP	152 (0%)	146 (0%)	298 (0%)		70 (3%)	
Vital signs						
Systolic blood pressure	125.9 ± 21.8	126.4 ± 21.8	126.2 ± 21.8	3575 (5%)	129.0 ± 13.6	3 (0%)
Diastolic blood pressure	68.9 ± 14.3	68.9 ± 14.3	68.9 ± 14.3	3612 (5%)	69.9 ± 13.6	3 (0%)
Heart rate	82.7 ± 16.8	82.1 ± 16.8	82.4 ± 16.8	5842 (8%)	83.3 ± 15.8	3 (0%)
Respiratory rate	19.0 ± 3.6	18.9 ± 3.4	18.9 ± 3.5	3876 (6%)	18.79 ± 3.72	2 (0%)
Oxygen saturation	97.8 ± 3.5	97.8 ± 3.5	97.8 ± 3.5	11,424 (17%)	96.9 ± 6.3	85 (4%)
Temperature (C)	36.8 ± 0.6	36.8 ± 0.5	36.8 ± 0.5	3796 (6%)	36.9 ± 0.5	3 (0%)
Laboratory findings						
Arterial PaCO_2_ (mmHg)	39.9 ± 10.6	39.9 ± 10.4	39.9 ± 10.5	58,426 (85%)	45.3 ± 12.8	1819 (74%)
Arterial PaO_2_ (mmHg)	134.0 ± 80.9	134.2 ± 81.2	134.1 ± 81.1	58,430 (85%)	80.9 ± 60.0	1862 (76%)
Arterial pH	7.4 ± 0.1	7.4 ± 0.1	7.4 ± 0.1	58,422 (85%)	7.4 ± 0.1	1819 (74%)
Hematocrit (%)	31.9 ± 6.4	32.0 ± 6.4	31.9 ± 6.4	4273 (6%)	32.6 ± 6.4	81 (3%)
Hemoglobin (g/dl)	10.6 ± 2.2	10.6 ± 2.2	10.6 ± 2.2	4301 (6%)	10.9 ± 2.1	81 (3%)
Platelet count (1000/μl)	227.7 ± 113.1	227.6 ± 113.9	227.7 ± 113.5	4306 (6%)	237.3 ± 101.9	85 (4%)
White blood count (1000/μl)	10.0 ± 7.8	10.0 ± 7.8	10.0 ± 7.8	4305 (6%)	9.7 ± 4.8	85 (4%)
Lactate (arterial or venous) (nM/L)	2.8 ± 3.2	2.1 ± 2.0	2.5 ± 2.8	67,076 (98%)	2.1 ± 1.3	1888 (77%)
Serum albumin (g/dl)	3.4 ± 0.7	3.4 ± 0.7	3.4 ± 0.7	22,145 (32%)	3.5 ± 0.7	1187 (48.3%)
Serum bicarbonate (meq/l)	23.8 ± 4.2	23.8 ± 4.2	23.8 ± 4.2	7058 (10%)	24.8 ± 4.2	254 (10%)
Serum calcium (mg/dl)	8.8 ± 0.8	8.8 ± 0.8	8.8 ± 0.8	12,099 (18%)	8.8 ± 0.8	254 (10%)
Serum chloride (meq/l)	102.1 ± 6.0	102.1 ± 6.0	102.1 ± 6.0	7055 (10%)	102.1 ± 5.7	254 (10%)
Serum creatinine (mg/dl)	1.7 ± 1.9	1.7 ± 1.9	1.8 ± 1.9	6681 (10%)	1.6 ± 1.9	254 (10%)
Serum glucose (mg/dl)	139.2 ± 73.8	139.0 ± 74.4	139.1 ± 74.1	6836 (10%)	145.0 ± 75.6	251 (10%)
Serum potassium (meq/l)	4.1 ± 0.6	4.1 ± 0.6	4.1 ± 0.6	6883 (10%)	4.3 ± 0.6	251 (10%)
Serum sodium (meq/l)	138.1 ± 5.0	138.1 ± 5.0	138.1 ± 5.0	6858 (10%)	140.5 ± 5.0	247 (10%)
Serum total bilirubin (mg/dl)	1.6 ± 3.7	1.5 ± 3.5	1.5 ± 3.6	21,489 (31%)	1.4 ± 3.5	1192 (48%)
Anion gap	15.9 ± 3.4	15.9 ± 3.4	15.9 ± 3.4	14,453 (21%)	18.08 ± 3.50	257 (11.3%)

Data presented as counts and percentages or mean ± standard deviation

APPROVE Accurate Prediction of Prolonged Ventilation, AVAPS average volume assured pressure support, BIPAP bilevel positive airway pressure, CPAP continuous positive airway pressure, FiO_2_ fraction of inspired oxygen, PaCO_2_ partial pressure of carbon dioxide, PaO_2_ partial pressure of oxygen, RASS Richmond Agitation-Sedation Scale

^*^Any use of indicated medication at selected time point

### Model development

**Fig. 1:**
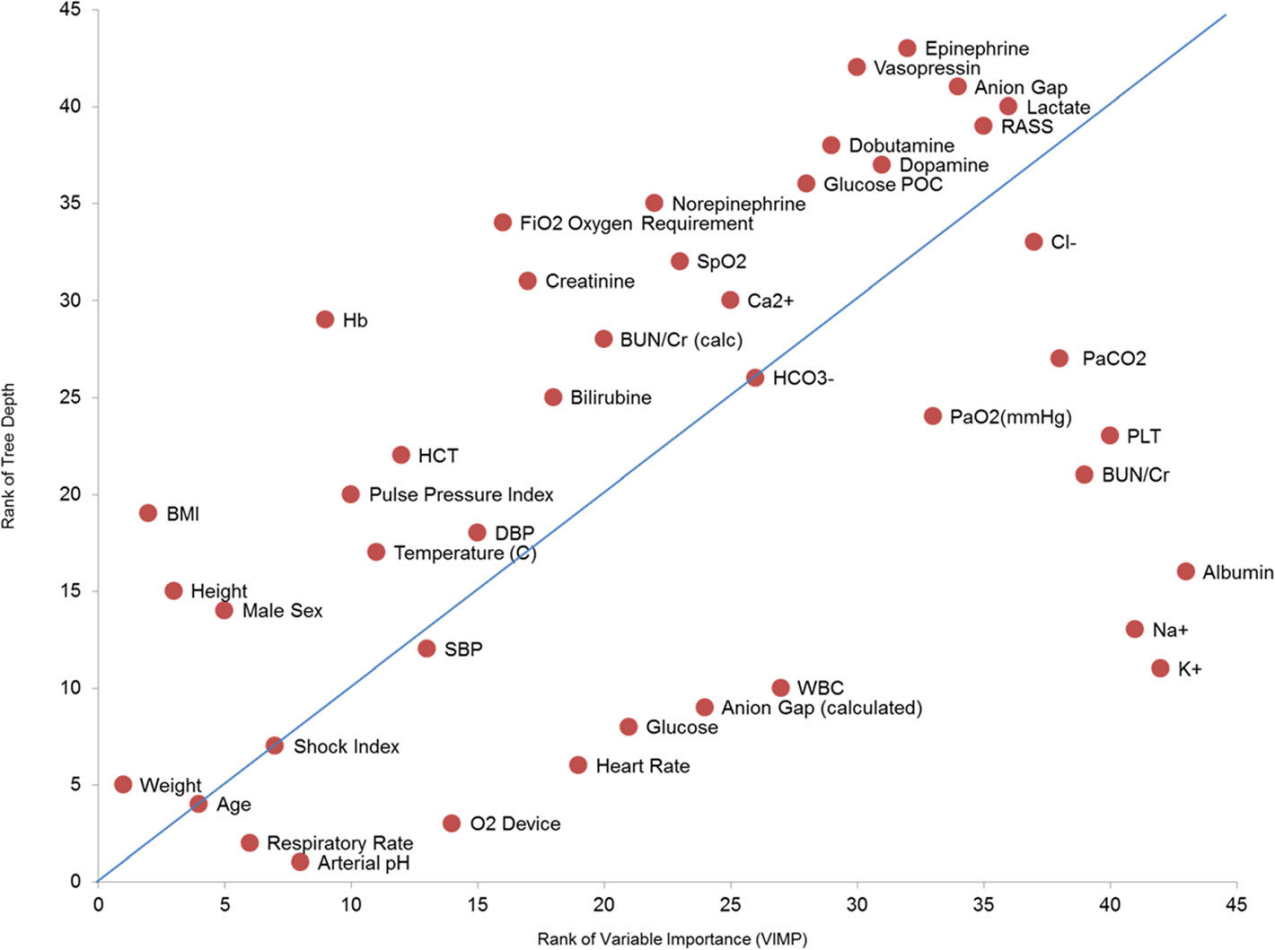
Relative importance of included variables. Tree depth is average depth at which variable is first used in a tree split, assuming that the most discriminatory variables will split the dataset earlier in trees at lower depths. Variable importance (VIMP) is a measure of how changes in the variable impact prediction error. The bigger the VIMP, the more impact the variable has on prediction. Variables having smaller depth are more discriminatory, and those with bigger importance have a greater impact on prediction. BMI body mass index, BUN blood urea nitrogen, DBP diastolic blood pressure, FiO2 fraction of inspired oxygen, Hb hemoglobin, HCT hematocrit, PaCO2 partial pressure of carbon dioxide, PaO2 partial pressure of oxygen, PLT platelet count, POC glucose point of care, RASS Richmond Agitation-Sedation Scale, SBP systolic blood pressure, SpO2 peripheral capillary oxygen saturation, WBC white blood cell count

In developing the model, the RF model was pruned at 200 trees as additional trees did not improve the predictive power of the model. The misclassification rate stabilized at 0.0243. The variables included in the model and the analysis of these variables by their importance in predicting events (variable importance) and average minimal depth among the 200 trees is presented in Fig. 1. Variables with low ranks for minimal depth and high ranks for variable importance are important for tree building and prediction.

### Model validation

**Fig. 2:**
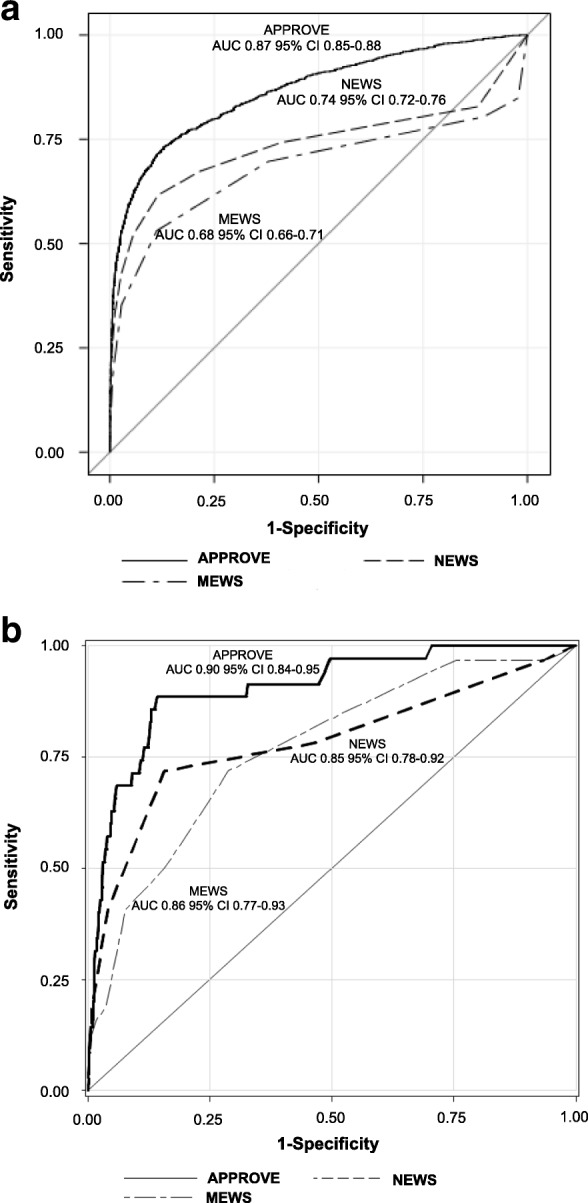
Area under the curve (AUCROC) for APPROVE, MEWS, and NEWS to predict for hospital mortality or intubation leading to mechanical ventilation > 48 h in retrospective 2013 validation cohort (**a**) and prospective 2017 validation hospital (**b**). APPROVE, MEWS and NEWS calculated at multiple random time points for each patient and evaluated for a qualifying event after score calculation. APPROVE Accurate Prediction of Prolonged Ventilation, CI confidence interval, MEWS Modified Early Warning Score, NEWS National Early Warning Score

**Fig. 3:**
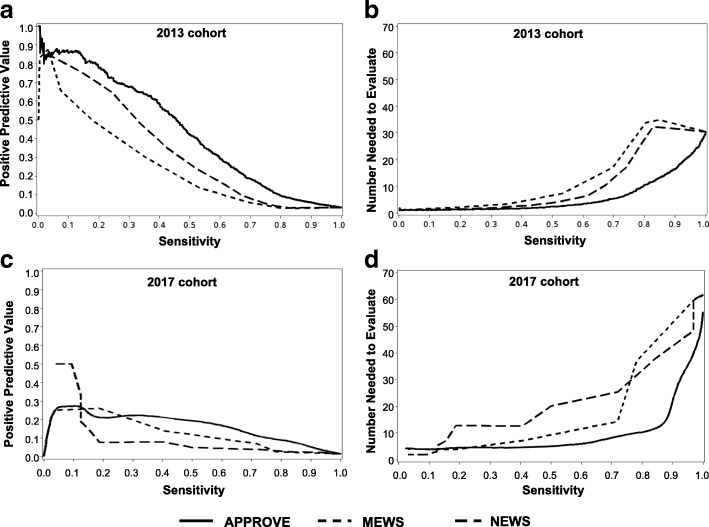
Positive predictive value (Fig. 3a for 2013 and Fig. 3c for 2017) and number of patients needed to be evaluated to identify one event (Fig. 3b for 2013 and Fig. 3d for 2017) as a function of sensitivity for APPROVE, MEWS and NEWS for the retrospective 2013 cohort (Fig. 3a and b) and the prospective 2017 cohort (Fig. 3c and d). Qualifying event is defined as hospital mortality or mechanical ventilation > 48 h

The AUROCs of APPROVE, MEWS, and NEWS to identify patients at high risk of death or MV  >= 48 h in the 2013 retrospective validation cohort are shown in Fig. 2a. In 2013, APPROVE had significantly better discrimination (AUROC 0.87, 95% CI 0.85–0.88)) compared to the MEWS (AUROC 0.68, 95% CI 0.66–0.71; *p* 0.0038) or NEWS (AUROC 0.74, 95% CI 0.72–0.76; *p* = 0.0055). However, the AUROC is limited in understanding how a prediction score may work in clinical practice. For clinical use, the sensitivity of the score to identify events must be balanced by the number of patients who will trigger the alert and the likelihood that an alert will predict the event. Figure 3 shows how the PPV and the number of patients needed to be evaluated to capture one event varies as a function of the sensitivity to detect an event if one was to alert clinicians when a patient’s score exceeds that value. Over a wide range of sensitivities, APPROVE had a higher PPV (Fig. 3a) while alerting on fewer patients in order to capture one event (Fig. 3b) as compared to the MEWS and NEWS.

To show how APPROVE may perform when used in the clinical setting when clinicians may be alerted whenever a patient’s score exceeds a certain threshold, Table 2 presents the sensitivity, specificity, PPV, NPV, and false positive rate (FPR) of APPROVE, MEWS and NEWS at different thresholds for alerting. In general, lower thresholds are associated with higher sensitivity but lower PPV (Table 2). The sensitivity and specificity of APPROVE to predict for any event subsequent to score calculation is good in 2013 (sensitivity 63–75% and specificity 83–92%). If alerts were sent for scores that exceed the threshold of 0.25, APPROVE would have a sensitivity of 63% (95% CI 60–66%) to detect an event, with approximately 1/5 alerted patients developing the event (PPV 21%, 95% CI 19–22%) and a FPR of 8%. In contrast, at a similar PPV for the MEWS at a threshold > 4, the sensitivity was only 16% (95% CI 13–18%). At a threshold for alerts of > 6 for NEWS, the PPV was similar at 22% (95% CI 19–25%) but, compared to APPROVE, the false positive rate was lower (1%) and sensitivity was also much lower at 16% (95% CI 13–18%).

**Table 2:** Performance of APPROVE in retrospective and prospective validation cohorts to predict for qualifying event at any time after score calculation

		APPROVE			MEWS			NEWS		
		Cutoff > 0.15	Cutoff > 0.20	Cutoff > 0.25	Cutoff > 4	Cutoff > 5	Cutoff > 6	Cutoff > 4	Cutoff > 5	Cutoff > 6
2013^a^	Sensitivity (95% CI)	0.75 (0.72–0.77)	0.69 (0.66–0.71)	0.63 (0.60–0.66)	0.16 (0.13–0.18)	0.08 (0.07–0.11)	0.04 (0.03–0.06)	0.28 (0.25–0.31)	0.22 (0.19–0.25)	0.16 (0.13–0.18)
	Specificity (95% CI)	0.83 (0.83–0.83)	0.88 (0.88–0.89)	0.92 (0.92–0.92)	0.98 (0.98–0.98)	0.99 (0.99–0.99)	0.99 (0.99–0.99)	0.94 (0.94–0.94)	0.97 (0.97–0.97)	0.99 (0.98–0.99)
	Positive predictive value (95% CI)	0.13 (0.12–0.14)	0.17 (0.16–0.18)	0.21 (0.19–0.22)	0.19 (0.16–0.22)	0.37 (0.30–0.44)	0.54 (0.42–0.66)	0.11 (0.10–0.13)	0.16 (0.14–0.18)	0.22 (0.19–0.25)
	Negative predictive value (95% CI)	0.99 (0.99–0.99)	0.99 (0.99–0.99)	0.99 (0.99–0.99)	0.98 (0.98–0.98)	0.98 (0.97–0.98)	0.98 (0.97–0.98)	0.98 (0.98–0.98)	0.98 (0.98–0.98)	0.98 (0.98–0.98)
	False positive rate (95% CI)	0.17 (0.17–0.17)	0.12 (0.11–0.12)	0.08 (0.08–0.08)	0.02 (0.02–0.02)	0.01 (0.01–0.01)	0.01 (0.01–0.01)	0.06 (0.06–0.06)	0.03 (0.03–0.03)	0.01 (0.01-0.01)
2017^b^	Sensitivity (95% CI)	0.89 (0.74–0.97)	0.69 (0.52–0.84)	0.64 (0.46–0.79)	0.67 (0.49–0.81)	0.39 (0.23–0.57)	0.19 (0.08–0.36)	0.67 (0.49–0.81)	0.47 (0.30–0.65)	0.39 (0.23–0.57)
	Specificity (95% CI)	0.85 (0.83–0.86)	0.91 (0.90–0.92)	0.95 (0.94–0.96)	0.86 (0.85–0.88)	0.96 (0.95–0.97)	0.99 (0.99–0.99)	0.75 (0.73–0.76)	0.86 (0.85–0.88)	0.93 (0.92–0.94)
	Positive predictive value (95% CI)	0.09 (0.06–0.12)	0.11 (0.07–0.16)	0.16 (0.11–0.23)	0.07 (0.05–0.11)	0.15 (0.08–0.23)	0.29 (0.13–0.51)	0.04 (0.03–0.06)	0.05 (0.03–0.08)	0.08 (0.05–0.14)
	Negative predictive value (95% CI)	0.99 (0.99–0.99)	0.99 (0.99–0.99)	0.99 (0.99–0.99)	0.99 (0.99–0.99)	0.99 (0.98–0.99)	0.99 (0.98–0.99)	0.99 (0.99–0.99)	0.99 (0.98–0.99)	0.99 (0.98–0.99)
	False positive rate (95% CI)	0.15 (0.13–0.16)	0.09 (0.08–0.10)	0.05 (0.04–0.06)	0.14 (0.12–0.15)	0.04 (0.03–0.04)	0.01 (0.00–0.01)	0.25 (0.23–0.27)	0.14 (0.12–0.15)	0.07 (0.06–0.08)

Qualifying event defined as death or intubation that leads to at least 48 h of mechanical ventilation

APPROVE Accurate Prediction of Prolonged Ventilation, CI confidence interval, MEWS Modified Early Warning Score, NEWS National Early Warning Score ale

^a^APPROVE calculated every time there are new data to predict for qualifying event any time in hospital after score calculation

^b^APPROVE calculated every 4 h after hospital admission to predict for qualifying event any time in hospital after score calculation

In the prospective 2017 cohort, APPROVE performed similarly to 2013 with an AUROC of 0.90 (95% CI 0.85–0.95) (Fig. 2). However, both the MEWS and NEWS performed better than they did in the 2013 cohort so that APPROVE was not significantly different from the MEWS and NEWS. The AUROC was 0.79 (95% CI 0.68–0.89) for the MEWS (*p* = 0.57 compared to APPROVE) and 0.77 (95% CI 0.67–0.86) for the NEWS (*p* = 0.76 compared to APPROVE). For sensitivities greater than 30%, the PPV was higher and the number of patients needing to be evaluated to capture one event was lower for APPROVE compared to the MEWS and NEWS (Fig. 3c and 3d). Similarly, Table 2 shows how the scores performed in the 2017 hospital at different thresholds for alerts. Again, the higher the threshold for each score, the lower the sensitivity, the higher the PPV, and the lower the false positive rate. At a threshold for alerts > 0.25, APPROVE had a sensitivity of 64% (95% CI 46–79%) similar to its performance in 2013 (sensitivity 63%, 95% CI 60–66%). With that threshold, approximately 16% of patients with an alert will develop an event (PPV 16%, 95% CI 11–23%) with a false positive rate of 5% (95% CI 4–6%). At a similar sensitivity with a threshold of > 4 for the MEWS and NEWS (67%, 95% CI 49–81%), the PPV was significantly lower (7%, 95% CI 5–11% for MEWS and 4%, 95% CI 3–6% for NEWS) and the false positive rate was significantly higher (14%, 95% CI 12–15% for MEWS and 25%, 95% CI 23–27% for NEWS) compared to APPROVE. Using a threshold for APPROVE of 0.20 shows similar results compared to MEWS or NEWS > 4.

Additional exploratory, sensitivity analysis was done to evaluate the performance of APPROVE for different events. In the first sensitivity analysis, we explored how well APPROVE, MEWS, and NEWS can predict for the individual events of hospital mortality, MV > 48 h, or MV of any duration (see Additional file 1: Figure S1). Although APPROVE was derived to predict for the combined outcome of hospital mortality or MV > 48 h, APPROVE was also able to predict for the individual events of hospital mortality (AUROC 0.91, 95% CI 0.90–0.93 in 2013; AUROC 0.93, 95% CI 0.89–0.97 in 2017), MV > 48 h (AUROC 0.79, 95% CI 0.77–0.80 in 2013; AUROC 0.86, 95% CI 0.73–0.94 in 2017), or MV of any duration (AUROC 0.77, 95% CI 0.76–0.79 in 2013; AUROC 0.80, 95% CI 0.67–0.92 in 2017). In 2013, this was significantly better than the MEWS and NEWS for hospital mortality but not significantly different from the MEWS and NEWS for MV. In 2017, APPROVE, MEWS and NEWS had similar AUROC for all of the individual events.

In another exploratory analysis, we explored the time from the first APPROVE score exceeding a given threshold to death or intubation leading to MV > 48 h (see Additional file 1: Table S3). The median time from first high APPROVE score to the primary composite event of mortality or MV > 48 h was 37.2 h (IQR 5.6–134.0), 29.5 h (IQR 4.5–121.2), and 26.8 h (IQR 4.5–111.2) at thresholds of 0.15, 0.20, and 0.25, respectively, in 2013. A longer time to event was seen in the 2017 validation hospital: 58.8 h (IQR 14.7–171.6), 58.8 h (IQR 13.1–190.6), and 68.6 h (IQR 10.7–193.8) at thresholds of 0.15, 0.20, and 0.25, respectively. As expected, the time to intubation is shorter, ranging from a median of 9.4 to 12.6 h in 2013 and from 7.4 to 32.8 h in 2017, depending on the threshold.

We also tried to investigate where patients were when they had an APPROVE score that would cross the threshold for potential alert. We were only able to do this in the 2017 prospective hospital as the ICU admission time was not reliable or accurate in the EHR systems of the 2013 hospitals. In the 2017 cohort, 85–91% of the patients were outside of the ICU when their APPROVE score crossed the threshold of 0.15, 0.20, or 0.25. For example, in the 188 patients with one or more APPROVE scores > 0.25 in the 2017 cohort, the first high score occurred outside of the ICU for 163 (89%) patients. Only 28% of these patients were ultimately transferred to an ICU and the median time from first high score to ICU transfer was 6 h (IQR 1.0–44.5).

## Discussion

Using random forest analysis, we were able to develop an EHR-based risk stratification tool that can identify patients at risk for in-hospital death or MV > 2 days. This was validated in a 2013 internal cohort and a 2017 prospective, external cohort in another hospital with AUROCs of 0.87 (95% CI 0.85–0.88) and 0.90 (95% CI 0.85–0.95), respectively. APPROVE in 2013 and 2017 had with similar sensitivity (63–89%), specificity (83–95%), NPV (99%), and manageable PPV (9–21%) and false positive rate (2–15%), depending on the threshold for alerts. In the 2013 retrospective validation cohort, APPROVE outperformed the MEWS and NEWS with a significantly higher AUROC, higher PPV, and fewer alerts needed to detect one event over a range of score sensitivities. But in 2017, APPROVE was not significantly different in performance as indicated by AUROC but had less false positive at similar sensitivities.

Most early warning scores aimed to predict for ICU admission, cardiac arrests, and/or death in the hospital. APPROVE differs from these prior approaches in that it aims to predict for mechanical ventilation or death in the hospital. For common conditions such as pneumonia, heart failure, COPD, pulmonary embolism, hip fractures, diabetic ketoacidosis, acute myocardial infarction, and strokes, ICU admission rates vary greatly by hospital and can depend as much on nonclinical hospital factors as on patient-level factors like diagnosis, severity of illness, or risk for mortality [12–17]. This is especially true for patients who are not intubated with lower severity of illness and risk of death (< 2%), which may constitute as much as 50% of the ICU admission to nonsurgical ICUs at some hospitals [15, 16]. Thus, patients with similar severity of illness and risk for death would be admitted to the ICU at some hospitals but not others, with no clear difference in outcomes. This raises the concern that early warning scores developed to predict for ICU admission at one hospital may perform variably in other settings depending on the ICU utilization rate and may not consistently correlate with mortality across different clinical settings. We intend APPROVE to be used in multiple hospitals. Acute respiratory failure requiring MV is consistently associated with increased mortality and morbidity and is a recognized criterion in guidelines for ICU admission [2, 4, 11, 18].

Although comparison to other published early warning scores is difficult as we aimed to predict for MV > 48 h or death in this study and prior studies predicted for different outcomes under different time frames and in different populations, the performance of the MEWS and NEWS in this study is similar to prior reports. The MEWS is reported to have an AUROC of 0.59–0.88 at predicting ICU transfer, cardiac arrest, or death within 24–48 h in different studies [19–23]. In one study, the MEWS had AUROC 0.68 for predicting ICU transfer and AUROC of 0.88 for predicting mortality within 24 h [19]. The NEWS has a reported AUROC of 0.65 for predicting in-hospital mortality at any time in sepsis while in another study it had an AUROC of 0.79 for in-hospital mortality and 0.83 for 24 h mortality among general medical patients [24, 25]. The performance of the MEWS and NEWS in our cohorts falls within the range of these prior reports (AUROC 0.78–0.93 for MEWS and 0.83–0.91 for NEWS for in-hospital mortality in the 2013 and 2017 cohorts (Additional file 1: Figure S1)).

Similarly, other EHR-derived early warning scores were derived to predict for different outcomes under different time frames. For example, the LAPS2 score had an AUROC of 0.88 for mortality but was derived to predict for hospital and 30-day mortality, and, as a result, utilizes outpatient comorbid data which are not available in most inpatient EHR systems [26]. An EHR-based score (eCART) had an AUROC of 0.77 for prediction of cardiac arrest, ICU transfer, or death and 0.93 for death [19] compared to APPROVE, with an AUROC of 0.87 and 0.90 for hospital mortality or MV > 48 h and 0.91 and 0.93 for death in 2013 and 2017, respectively [27]. The Rothman Index predicted for hospital mortality at an AUROC of 0.93 in predicting mortality within 24 h [28] while APPROVE has an AUROC of 0.91 in 2013 and 0.93 in 2017 for death and a median time to death of about 3–6 days.

In 2013, APPROVE outperforms the MEWS and NEWS by AUROC, sensitivity, and relative balance between sensitivity of the score and the PPV or number of patients alerted to identify one event. While APPROVE’s performance in 2017 is very similar to that in 2013, APPROVE in the 2017 cohort was not significantly different in AUROC compared to the MEWS and NEWS. This is partly due to the sample size in the 2017 cohort, which limits the power to detect a difference. But it may also be due to improved performance of the MEWS and NEWS in the 2017 prospective cohort. This improvement may be due to differences between patients in the two cohorts and how the scores were calculated retrospectively vs prospectively. In the 2013 retrospective cohort, APPROVE, MEWS and NEWS were calculated on all adult admissions whenever there were data available, from the initial presentation to the emergency department (ED) to hospital discharge. In the 2017 prospective hospital, the calculation of APPROVE and the other scores started after the placement of a hospital admission order at the next scheduled time (00:00, 04:00, 08:00, 12:00, 16:00, 20:00). Patients who presented to the ED and were intubated or died before hospital admission and calculation of any APPROVE score were excluded from the 2017 analysis. Indeed, 33 patients or 49% of all patients with an event in the prospective hospital were excluded from the analysis. However, no risk assessment model is likely to be useful in those patients who present with respiratory failure requiring intubation on or shortly after presentation to the ED. So, the exclusion of these patients in the 2017 analysis may improve the performance of any prediction score while the inclusion of similar patients in the 2013 cohort may have limited the ability of the score to predict intubation. Nevertheless, it is likely that APPROVE may best be used for patients admitted to the hospital ward rather than for patients on initial presentation to the hospital or patients in the ED with short ED length of stays.

However, the AUROC should not be the only indicator of how well a risk assessment tool may be used in clinical practice. A clinically useful tool must also balance the sensitivity to detect an event with the number of patients who will trigger an alert and develop the event. Alerting on too many patients who may not develop the event can lead to inefficient allocation of resources and contribute to alert fatigue. Compared to the MEWS and NEWS, APPROVE presented a higher percentage of alerted patients who subsequently developed the event while alerting on fewer patients in order to capture one event (Fig. 3). If alerts are sent when scores exceed the threshold of 0.25 in the 2017 cohort, APPROVE had a sensitivity of 64% and specificity of 95% with approximately 16% of alerted patients developing the event at a false positive rate of 5%. At a similar sensitivity of 67% for MEWS or NEWS > 4, the PPV was significantly lower (7%, 95% CI 5–11% for MEWS and 4%, 95% CI 3–6% for NEWS) and the false positive rate was higher (14%, 95% CI 12–15% for MEWS and 25%, 95% CI 23–27% for NEWS) than APPROVE. Similar results are seen in the 2013 cohort for alerts at thresholds > 0.25.

An automated risk prediction score offers a potential advantage if it can be incorporated into the clinical setting for prospective identification of at-risk patients. Application of prediction scores in the clinical setting requires linkage to some action (efferent arm) which could include notification of the patient’s providers with or without triggering a rapid response. Such a first-line clinician filter will be needed as the use of APPROVE will require a balance of early detection against false alerts. Lowering the cutoff value will result in a higher sensitivity but at the cost of a greater number of patient alerts at a lower event rate. As APPROVE is a continuous score, the cutoff value for alerts can be tailored for an acceptable alert rate and intervention depending on the risk of the intervention.

In the 2017 cohort, clinicians were alerted when their patients APPROVE score exceeded 0.25. This may have influenced their behavior including their decision to intubate.

As the majority of alerts for APPROVE occur in non-ICU patients at a median of 26–68 h prior to death or intubation leading to MV > 48 h or 7.4–33 h before intubation, there may be sufficient time for potential interventions. The triggering of APPROVE could activate enhanced monitoring, rapid response, or interventions that could prevent progression of respiratory failure. Examples of such interventions include earlier administration of or escalation in diuretics, bronchodilator therapy, high-flow nasal cannula, or noninvasive ventilation, as appropriate. It may also mean earlier re-evaluation of patient response to noninvasive ventilation. Since mechanical ventilation is a life-saving intervention that will be unavoidable in some of these patients, early and timely implementation of best practices such as low tidal volume in ARDS may lead to better outcomes [29–31]. In addition, APPROVE would also allow for earlier discussion of goals of care, a component of the Choosing Wisely Campaign that is increasingly being done emergently during a RRT [32, 33]. In the prospective hospital, clinicians were alerted if their hospital ward patient’s APPROVE score exceeded a threshold of 0.25. Additional studies will be needed to determine whether linking automated prediction scores to alerts will result in improved clinical outcomes.

Our study has several limitations. As discussed, APPROVE was not designed to predict for patients with short-term MV. In the EHR system of the study hospitals, MV can be reliably identified but not the reason for intubation. We focused on MV > 48 h to avoid those patients intubated electively for a procedure or surgery. Nevertheless, APPROVE was still able to identify patients who required MV of any duration (AUROC 0.77 and 0.80 in 2013 and 2017 cohort, respectively), although it was no better than the MEWS or NEWS. APPROVE was also not designed to identify less severely sick patients with isolated nonpulmonary organ failure such as sepsis that may require vasopressors without the need for MV. However, respiratory dysfunction and failure is among the most common organ failure in septic shock, with up to 84% of septic shock patients requiring MV, which contributes greatly to the risk for mortality in sepsis [34, 35]. This score was created in hospitals with a comprehensive EHR, which may be difficult to replicate in a community hospital with more limited EHR systems. We lacked accurate data on ICU admission time in 2013 to determine how often alerts occur outside of the ICU. However, data from the 2017 cohort indicate that most alerts (> 85%) occur outside of the ICU with a median of 6 h prior to ICU admission. In the 2017 hospital, APPROVE was calculated after a hospital admission order was placed at 4-h intervals, which missed a significant number of patients who died or were intubated prior to any score calculation. We are planning to move the trigger for score calculation to ED triage and to real-time score calculation. We plan to evaluate how the performance of APPROVE may change with more timely calculation of the score. Any prediction model has a risk of changing in sensitivity over time due to the “effect of training”. However, validation of APPROVE in 2017 still showed good performance. The sample size in 2017 was smaller than in 2013 with a lower event rate which may have reduced the power to detect a statistically significant difference in performance between scores. Nevertheless, the similarity in performance of APPROVE in the two cohorts is reassuring even with a smaller sample size. Lastly, our algorithm should be tested for accuracy, impact on decision-making, and validation for patient-centered outcomes in a wide variety of clinical settings and patient cohorts.

Our study has several strengths. APPROVE was derived using random forests, a nonparametric, machine learning approach that has been shown to outperform other methods [20]. Additionally, APPROVE uses random forest imputation methods for missing data, which outperforms most other methods of missing data imputation [36]. APPROVE was compared to the NEWS and MEWS, as they are commonly used currently as early warning scores. Most prior studies derived and validated their EHR-based scores at one hospital. APPROVE was derived and validated in a total of five hospitals with three different EHR systems. Nevertheless, despite the differences in the EHR, patient population, event rates, and frequency of calculation of the APPROVE score in 2013 and 2017, the sensitivity, specificity, PPV, and NPV were similar. Lastly, APPROVE was validated prospectively which better represents how it may perform in a real-world clinical setting and illustrates the feasibility of operationalizing predictive analytics at the bedside.

## Conclusions

We have developed a risk stratification tool to identify patients in the hospital at risk of death or acute respiratory failure requiring MV > 48 h that performs better than the MEWS and NEWS while alerting on fewer patients. After validation in a multicenter cohort in 2013, APPROVE was integrated into the EHR for automated calculation every 4 h in 2017 for prospective validation. The performance of APPROVE was similar between the prospective and retrospective validation cohort, indicating the feasibility of prospective integration of predictive analytics into the hospital record system for the identification of patients at high risk for respiratory failure or death. Additional studies will be needed to determine whether linking automated prediction scores to alerts will result in improved clinical outcomes.

## Additional file

Additional file 1: **Table S1.** Variables selected for model. **Table S2.** APPROVE performance in derivation cohort to predict for event within 48 h of score. **Table S3.** Median (interquartile range) time to event from when APPROVE first exceeds threshold during hospitalization to primary event of death or intubation that leads to MV > 48 h, or to individual events of hospital death, or intubation leading to MV > 48 h in 2013 and 2017 validation cohorts. For composite qualifying event, earliest event (death or intubation leading to MV> 48 h) was considered the time of the event. **Figure S1.** Area under the curve (AUROC) for APPROVE, MEWS, and NEWS to predict for hospital mortality (**a**), mechanical ventilation (MV) > 48 h (**b**) and MV of any duration (**c**) in retrospective 2013 validation hospital and (**d, e, f**, respectively) prospective 2017 validation hospital. APPROVE, MEWS and NEWS calculated at multiple random time points for each patient and evaluated for a qualifying event after score calculation. (DOCX 141 kb)
