# Supplementary material for: Multicenter derivation and validation of an early warning score for acute respiratory failure or death in the hospital
Source: Crit Care. 2018 Oct 30;22:286. doi: 10.1186/s13054-018-2194-7 (PMC6206729; doi:10.1186/s13054-018-2194-7)
Supplement: Supplementary file 1 — Table S1. Variables selected for model. Table S2. APPROVE performance in derivation cohort to predict for event within 48 h of score. Table S3. Median (interquartile range) time to event from when APPROVE first exceeds threshold during hospitalization to primary event of death or intubation that leads to MV > 48 h, or to individual events of hospital death, or intubation leading to MV > 48 h in 2013 and 2017 validation cohorts. For composite qualifying event, earliest event (death or intubation leading to MV> 48 h) was considered the time of the event. Figure S1. Area under the curve (AUROC) for APPROVE, MEWS, and NEWS to predict for hospital mortality (a), mechanical ventilation (MV) > 48 h (b) and MV of any duration (c) in retrospective 2013 validation hospital and (d, e, f, respectively) prospective 2017 validation hospital. APPROVE, MEWS and NEWS calculated at multiple random time points for each patient and evaluated for a qualifying event after score calculation. (DOCX 141 kb) [file 13054_2018_2194_MOESM1_ESM.docx]

**SUPPLEMET**

**Table S1 Variable, selected for the model**

| baseline demographics | age (years), sex, height (cm), weight (kg), Body Mass Index (kg/m2) |
| --- | --- |
| vital signs | heart rate (beats per minute), respiratory rate (per minute), systolic blood pressure (mmHg), diastolic blood pressure (mmHg), temperature (C^0^), pulse pressure^1^* (relative difference between the systolic and diastolic pressure), shock index^2^*. |
| laboratory data | anion gap (meg/l)*, arterial PaCO2 (mmHg), arterial PaO2 (mmHg), arterial pH (mmHg), BUN/Creatinine ratio, hematocrit (%), hemoglobin (g/dl), lactate of an arterial or venous blood gas (nm/l), serum albumin (g/dl), serum anion gap (meg/l), serum bicarbonate (meq/l), serum blood urea nitrogen (bun) (mg/dl), serum calcium (mg/dl), serum chloride (meq/l), serum creatinine (mg/dl), serum glucose (mg/dl), serum potassium (meq/l), serum sodium (meq/l), serum total bilirubin (mg/dl), white blood count (10k/μl) |
| hospital intervention/assessment | vasopressor use** (dobutamine, dopamine, epinephrine, norepinephrine, vasopressin), oxygen delivery device (ranging from room air to high flow nasal cannula and BiPAP), Richmond agitation-sedation scale (RASS) value |

* – variables, calculated with the variables with the closest time stamps to the initial source data;

** – the vasopressor use was defined as 1 if the patient had any dose of vasopressors at specific time-stamp and 0 otherwise.

1 - pulse pressure – relative difference between the systolic and diastolic pressure

2 - shock index – heart rate value divided by systolic blood pressure value (Allgower M, Burri C. ["Shock index"]. *Dtsch Med Wochenschr.* 1967;92(43):1947-1950).

**Table S2 APPROVE performance in the derivation cohort to predict for event within 48 hours of score**

| **APPROVE Score Cut Point** | **Sensitivity** | **Specificity** | **PPV** | **NPV** |
| --- | --- | --- | --- | --- |
| 0.01 | 1.00 (1.00-1.00) | 0.47 (0.47-0.48) | 0.05 (0.05-0.05) | 1.00 (1.00-1.00) |
| 0.02 | 1.00 (1.00-1.00) | 0.69 (0.69-0.70) | 0.09 (0.08-0.09) | 1.00 (1.00-1.00) |
| 0.03 | 1.00 (1.00-1.00) | 0.81 (0.81-0.82) | 0.13 (0.13-0.14) | 1.00 (1.00-1.00) |
| 0.04 | 1.00 (1.00-1.00) | 0.88 (0.87-0.88) | 0.19 (0.19-0.19) | 1.00 (1.00-1.00) |
| 0.05 | 1.00 (1.00-1.00) | 0.92 (0.91-0.92) | 0.26 (0.25-0.26) | 1.00 (1.00-1.00) |
| 0.10 | 1.00 (1.00-1.00) | 0.98 (0.98-0.98) | 0.58 (0.58-0.59) | 1.00 (1.00-1.00) |
| 0.15 | 0.99 (0.99-0.99) | 0.99 (0.99-0.99) | 0.79 (0.78-0.79) | 1.00 (1.00-1.00) |
| 0.20 | 0.81 (0.81-0.82) | 1.00 (1.00-1.00) | 0.87 (0.87-0.87) | 0.99 (0.99-1.00) |
| 0.25 | 0.59 (0.58-0.59) | 1.00 (1.00-1.00) | 0.91 (0.91-0.92) | 0.99 (0.99-0.99) |
| 0.30 | 0.45 (0.44-0.45) | 1.00 (1.00-1.00) | 0.94 (0.94-0.95) | 0.98 (0.98-0.99) |

Data are presented mean and 95% confidence interval

PPV – Positive Predictive Value; NPV – Negative Predictive Value

**Table S3:** Median (Interquartile range) of time to event from when APPROVE first exceed the threshold during the hospitalization to the primary event of death or intubation that leads to mechanical ventilation greater than 48 hours, or to the individual events of hospital death, or intubation leading to mechanical ventilation greater than 48 hours in the 2013 and 2017 validation cohorts. For the composite qualifying event, the earliest of the event (death or intubation leading to mechanical ventilation> 48 hours) was considered the time of the event.

| **Event** | **Median time in hours (Interquartile range) from APPROVE score first exceeding threshold to event** | | | | | |
| --- | --- | --- | --- | --- | --- | --- |
|  | **Threshold for APPROVE in Retrospective 2013 Cohort (N=34,388)** | | | **Threshold for APPROVE in Prospective 2017 Cohort (N=2258)** | | |
|  | **>0.15** | **>0.20** | **>0.25** | **>0.15** | **>0.20** | **>0.25** |
| Death or Mechanical Ventilation > 48 hours | 37.2  (5.6-134.0) | 29.5  (4.5-121.2) | 26.8  (4.5-111.2) | 58.8 (14.7,171.6) | 58.8 (13.1,190.6) | 68.6 (10.7,193.8) |
| Death | 98.8  (22.1-224.2) | 79.9  (16.5-216.4) | 76.7  (15.2-202.0) | 145.4 (32.1,236.9) | 149.7 (47.4,234.9) | 148.6 (46.3,235.4) |
| Mechanical Ventilation > 48 | 12.6  (3.1,-62.0) | 10.3  (2.6-49.1) | 9.4  (2.5-43.8) | 32.8 (3.7,68.9) | 8.3 (0.8,52.1) | 7.4 (0.5,60.5) |

**Online Data Supplement Figure 1:** Area under the curve (AUROC) for APPROVE, MEWS, and NEWS to predict for hospital mortality (Fig. 1a), mechanical ventilation (MV) > 48 hours (Fig. 1b) and MV of any duration (Fig. 1c) in the retrospective 2013 validation and the prospective 2017 validation hospital (Fig. 1 d,e,f, respectively). APPROVE, MEWS and NEWS were calculated at multiple random time points for each patient and evaluated for a qualifying event after score calculation.


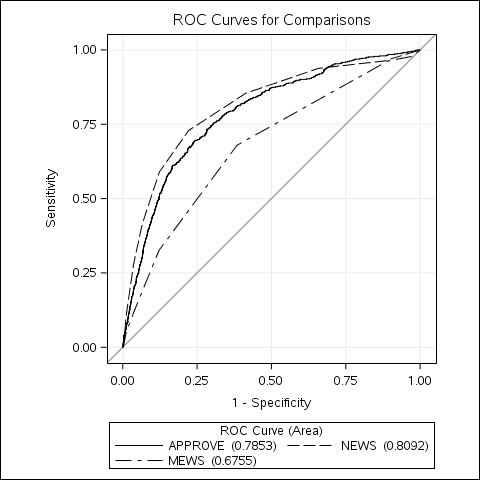

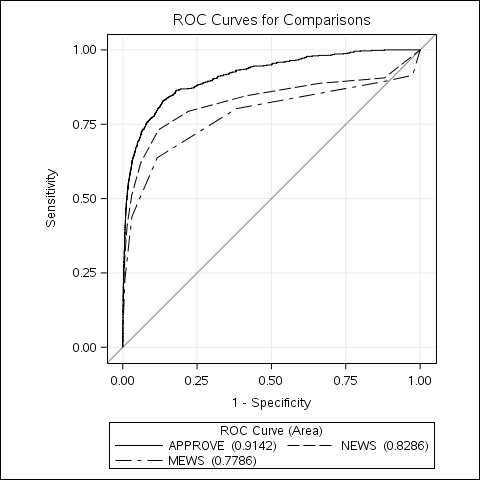


**Fig. 1b:** MV > 48 hours in 2013

**Fig. 1a:** Hospital Mortality in 2013

**Fig. 1c:** MV of Any Duration in 2013

Sensitivity


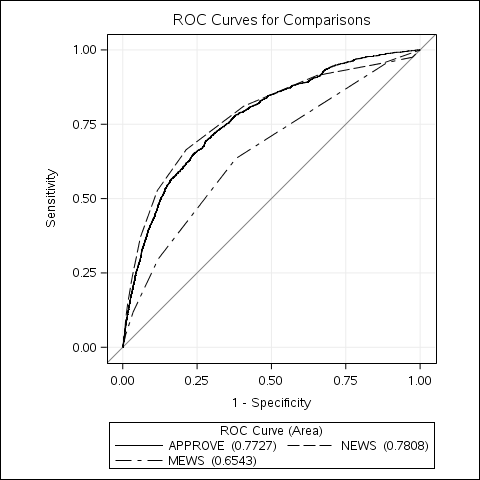


|  | Any MV |
| --- | --- |
| APPROVE | 0.77 (0.76,0.79) |
| MEWS | 0.65 (0.64,0.67) |
| NEWS | 0.78 (0.77,0.79) |

|  | MV > 48 hours |
| --- | --- |
| APPROVE | 0.79 (0.77,0.80) |
| MEWS | 0.68 (0.66,0.70) |
| NEWS | 0.81 (0.79,0.83) |

|  | Hospital Mortality |
| --- | --- |
| APPROVE | 0.91 (0.90,0.93) |
| MEWS | 0.78 (0.75,0.80) |
| NEWS | 0.83 (0.81,0.85) |

1-Specificity

1-Specificity

1-Specificity

**Fig. 1f:** MV of Any Duration in 2013

**Fig. 1e:** MV > 48 hours in 2017

**Fig. 1d:** Hospital Mortality in 2017

|  | Hospital Mortality |
| --- | --- |
| APPROVE | 0.93 (0.89,0.97) |
| MEWS | 0.93 (0.85,0.98) |
| NEWS | 0.91 (0.85,0.96) |

|  | MV > 48 hours |
| --- | --- |
| APPROVE | 0.86 (0.73,0.94) |
| MEWS | 0.79 (0.62,0.92) |
| NEWS | 0.81 (0.68,0.93) |

|  | Any MV |
| --- | --- |
| APPROVE | 0.80 (0.67,0.92) |
| MEWS | 0.72 (0.57,0.84) |
| NEWS | 0.73 (0.58,0.86) |

1-Specificity

1-Specificity

1-Specificity

Sensitivity
